# Dissecting the transcriptome in cardiovascular disease

**DOI:** 10.1093/cvr/cvab117

**Published:** 2021-03-23

**Authors:** Emma L Robinson, Andrew H Baker, Mairi Brittan, Ian McCracken, G Condorelli, C Emanueli, P K Srivastava, C Gaetano, T Thum, M Vanhaverbeke, C Angione, S Heymans, Y Devaux, T Pedrazzini, F Martelli, EU-CardioRNA COST Action CA17129

**Affiliations:** 1 Department of Cardiology, Cardiovascular Research Institute Maastricht (CARIM), Universiteitssingel 50, 6229 Maastricht University, Maastricht, The Netherlands; 2 The Division of Cardiology, School of Medicine, University of Colorado Anschutz Medical Campus, Aurora, CO, USA; 3 Centre for Cardiovascular Science, Queen’s Medical Research Institute, University of Edinburgh, 47 Little France Crescent, Edinburgh, EH16 4TJ, UK; 4 Humanitas Research Hospital, Humanitas University, Via Manzoni 113, Rozzano, MI 20089, Italy; 5 Imperial College, National Heart and Lung Institute, Hammersmith campus, Du Cane Road, London W12 0NN, UK; 6 Laboratorio di Epigenetica, Istituti Clinici Scientifici Maugeri IRCCS, Via Maugeri 4, Pavia 27100, Italy; 7 Hannover Medical School, Institute of Molecular and Translational Therapeutic Strategies (IMTTS), Carl-Neuberg-Straße 1 30625 Hannover, Germany; 8 UZ Gasthuisberg Campus, KU Leuven, Herestraat 49 3000 Leuven, Belgium; 9 Department of Computer Science and Information Systems, Teesside University, Middlesbrough, TS4 3BX, UK; 10 Cardiovascular Research Unit, Department of Population Health, Luxembourg Institute of Health, 1A-B, rue Thomas Edison, L-1445 Strassen, Luxembourg; 11 Experimental Cardiology Unit, Division of Cardiology, Department of Cardiovascular Medicine, University of Lausanne Medical School, 1011 Lausanne, Switzerland; 12 Molecular Cardiology Laboratory, IRCCS-Policlinico San Donato, Piazza Edmondo Malan, 2, 20097 San Donato, Milan, Italy

**Keywords:** Transcriptomics, Non-coding RNAs, Methodology standardisation, Translational cardiovascular research

## Abstract

The human transcriptome comprises a complex network of coding and non-coding RNAs implicated in a myriad of biological functions. Non-coding RNAs exhibit highly organized spatial and temporal expression patterns and are emerging as critical regulators of differentiation, homeostasis, and pathological states, including in the cardiovascular system. This review defines the current knowledge gaps, unmet methodological needs, and describes the challenges in dissecting and understanding the role and regulation of the non-coding transcriptome in cardiovascular disease. These challenges include poor annotation of the non-coding genome, determination of the cellular distribution of transcripts, assessment of the role of RNA processing and identification of cell-type specific changes in cardiovascular physiology and disease. We highlight similarities and differences in the hurdles associated with the analysis of the non-coding and protein-coding transcriptomes. In addition, we discuss how the lack of consensus and absence of standardized methods affect reproducibility of data. These shortcomings should be defeated in order to make significant scientific progress and foster the development of clinically applicable non-coding RNA-based therapeutic strategies to lessen the burden of cardiovascular disease.

## 1. Introduction

The completion of the human genome project in 2003—an international joint public and private effort—is a date that will go down in history.^1–3^ Sequencing of all 46 chromosomes in the human genome revealed that we possess less than 25% of the predicted 100,000 protein-coding genes, a surprisingly similar number of protein-coding genes to that in the nematode worm. 

We now know that the human transcriptome is much more complex than previously thought. The advent of deep sequencing tools of the last 15 years has revealed a complex network of at least 10,000 long non-coding RNAs, encoded in a myriad of genomic contexts.^4^

Non-coding RNAs are functional RNA molecules that lack protein-coding potential. Only a subset have so far been characterized, and many exhibit high spatial and temporal expression patterns and are emerging as key regulators of differentiation, development, homeostasis, and disease pathology, including in cardiovascular disease (CVD).

Delineating the transcriptome changes in cardiac cell types and defining their regulation in disease remodelling could not only enable us to further understand the cellular and molecular processes underlying pathology, but also provides the opportunity to select new, specific and more effective therapeutic targets to treat CVD.

This review describes the challenges in dissecting and understanding transcriptome remodelling in CVD, focusing on non-coding genes. We will then discuss strategies being implemented to overcome these confounding factors. Finally, we review hurdles yet to overcome to improve reproducibility of data, convincingly delineate the cellular and molecular processes underlying cardiovascular pathology and catalyse translational impact.

MicroRNAs (miRNAs) are 21- to 23-nt-long short, single-stranded non-coding RNAs involved in post-transcriptional regulation of gene expression through binding to complementary sites predominantly in the 3’ untranslated regions (UTR) of pre-messenger RNA (mRNA, protein-coding).^5^ This binding event represses translation of the mRNA, often targeting it for degradation. At least 60% of protein-coding genes are miRNA targets. Each miRNA can negatively regulate a number of different mRNAs and each of the miRNA-regulated protein-coding genes can be targeted by a number of miRNAs. This pleiotropic property of miRNAs has its advantages and disadvantages with regards to therapeutic potential. Complex diseases, including CVD, are most commonly multi-factorial and multi-genic, a consequence of multiple genetic and non-genetic factors. A miRNA often targets a number of mRNAs that converge on common biological pathways and processes. A large number of miRNAs are dysregulated in CVD, in the heart or blood, identified through pre-clinical and translational studies.^6^

MiRNA therapy has gained a lot of attention due to its specific targetability using chemically modified antisense technology, enabled by the inherent complementary base-pairing properties of nucleic acids. Whilst a handful of antisense therapies have been approved to date by the US Food and Drug Administration (FDA) targeting messenger RNAs, no miRNA drugs have thus far made it onto the market. Contributing to the low rate of translation to the clinic is a lack of consensus methodologies and experimental approaches. Pre-clinical work in large animal models has preceded clinical trials, which are currently ongoing.

Another facet of miRNA research in biomedical science is as circulating predictive, diagnostic, and prognostic biomarkers of disease. Blood, plasma, serum, and other biological fluids such as urine and tear drops have been found to be rich in RNA, either through release into the circulation from necrotic or apoptotic cells or having been actively secreted in microvesicles such as exosomes.^7^

Long non-coding RNAs (lncRNAs) are defined as transcribed non-protein-coding RNA molecules longer than 200 nt in length. LncRNA genes can overlap protein-coding genes, be located in an antisense orientation to protein-coding genes, intronic or intergenic (also known as long intervening noncoding RNAs (lincRNAs).^8^ In general, lncRNAs are less well conserved than protein-coding genes and exhibit a greater degree of temporal expression patterns and cell-type specificity. Their biological functions are as heterogeneous and widespread as proteins. LncRNAs are emerging as key mediators of differentiation, development, homeostasis, ageing, and disease.

Among lncRNAs, enhancer RNAs (eRNAs) describe a family of non-coding transcripts generated from enhancer regions of annotated genes that have a cis-regulatory role in transcription. Enhancer RNAs have been identified as regulators in cardiomyocyte(CM) differentiation development and homeostasis as well as being altered in response to pathological stimuli.^9–11^

A further recently discovered class of non-coding RNAs are the circular RNAs (circRNAs). CircRNAs are single-stranded and created by back splicing of 3’-5’ linear coding or non-coding RNAs, forming covalently closed loops.^12^ Originally thought to be a result of mis-splicing, circRNAs are now emerging as a major class of regulatory RNA molecules in disease and development. More than 30,000 circRNAs are now described in the human genome and diverse biological functions described and predicted including microRNA sponging and sequestration and protein binding affecting their stability.^13^

CircRNAs are being examined not only for their intracellular activity but also as stable and specific circulating biomarkers reflecting cardiac dysfunction and pathology. The stability of circRNAs in the circulation exceed that of linear RNAs and their diagnostic value for CVD has started to be evaluated.^14^^,^^15^

We are now aware that a significant number of lncRNAs and unannotated transcripts originally defined as purely non-coding have short, translated open reading frames (sORFs).^16^ A number of strategies, including conservation analysis, have revealed micropeptides with important functional roles in biological processes. Accordingly, a recent sophisticated high-throughput examination of the ‘translatome’ in the healthy and diseased human heart by ribosome profiling revealed a vast number of potentially translated sORFs, including within 77 lincRNAs and 64 antisense transcripts.^17^ Validation and biological relevance of these translatable regions remains to be elucidated.

## 2. RNA sources and RNA integrity

Whilst some aspects of CVD can be modelled in animals, due to our lack of understanding along with practicalities, biological differences, and multi-factorial aetiologies, human CVD can never be perfectly emulated in pre-clinical models. Thus, to confirm the translational potential of findings in cellular and animal studies, human specimen can be used to show conservation of transcriptome analyses.

Human specimens used in CVD research include, but are not limited to, liquid biopsies such blood, plasma, serum, buffy coat, isolated blood cells, and solid tissues, such as cardiac biopsies, explanted cardiac tissue from heart transplant patients and hearts extracted from cadavers of victims of sudden death (so-called ‘donor’ hearts). Guidelines for standardized sample collection and storage are not well established, which leads to variability in the quality of RNA available.

Isolation of good quality, intact RNA is required for techniques used for RNA and gene expression analysis including RTqPCR, RNA-sequencing (RNA-seq) and northern blotting. RNA integrity can be assessed qualitatively by running on a denaturing gel (e.g. with formaldehyde) and stained with a nucleic acid stain such as ethidium bromide. Intact RNA will give sharp, clean 28S and 18S ribosomal RNA (rRNA) bands, with the 28S approximately twice as intense as the 18S band. Degraded RNA will lack these sharp rRNA bands with a 2:1 ratio and have a smear-like appearance. A faint smear may appear between 1–2 kb, containing other RNAs such as mRNAs and lncRNAs. With the knowledge that at least 80% of the total cellular RNA is rRNA, RNA integrity can additionally be assessed with the knowledge of the mass of total RNA loaded and the rRNA bands compared against an RNA ladder of known marker size and mass. Elementary variations of this method have been devised to avoid the use of hazardous denaturing substances and improve resolution.^18^^,^^19^ Whilst gels offer a fast and simple means to assess RNA integrity with basic equipment used in molecular biology, even the latest nucleic acid gel imaging technologies and analysis tools are considered semi-quantitative at best.

The gold standard for assessing RNA integrity is the 2100 Bioanalyzer (Agilent). This device provides an automated electrophoresis strategy using microfluidics technology for RNA integrity analysis based on the relative rRNA species intensity. A range of microfluidics chips are available for different concentrations of RNA sample, with as little as 50 pg required by the Pico RNA analysis products. The 2100 Bioanalyzer calculates an RNA Integrity Number (RIN), which is an algorithm for robust and reliable assignment of quality values to RNA samples.^20^ RIN calculation still includes 28S and 18S ratio as well as any RNA species detected between the 18S and smaller 5S band. An alternative to the 2100 Bioanalyzer, also by Agilent, is the TapeStation, which also uses capillary electrophoresis but requires slightly higher volumes and concentrations of RNA (at least 2 ul of ≥ 5 ng/ul).

Of note, these methods to assess RNA integrity are based on ribosomal RNAs. This is not suitable for assessing the quality of nuclear RNA or polyA RNA preparations, which are depleted of rRNA. Some semi-quantitative methodologies have been suggested for assessment of polyA RNA samples.^21^

There is a lack of consensus regarding the minimal RIN value that should be used for accurate execution of RNA analysis. Moreover, different techniques for RNA analysis may be able to withstand different RNA integrity thresholds to be accurate and reproducible. For example, cDNA library synthesis requires initial RNA degradation into fragments either through heating or enzymatic digestion. Many researchers and next-generation sequencing (NGS) technology companies and facilities arbitrarily state a minimum RIN value of 7 or 8 for deep RNA-sequencing. In the context of clinical samples in translational cardiovascular research, this minimum RIN is often unrealistic, with the example of fluid biopsies remaining on the bench at room temperature while the cardiologist tends to the patient.

In the context of transcriptome analysis, a number of confounding factors can affect the outcome of research findings. In particular, the quality and integrity of RNA, determined by a number of pre-analytical variables along with sampling and storage methods.^22^*Table 1* provides an overview of confounding factors and pre-analytical variables affecting RNA integrity and outcomes of gene expression analysis.

**Table 1:** Pre-analytical variables in translational cardiovascular research

Variable	Applicability to translational cardiovascular research	References
(1) Post-mortem interval	The time between death and sample collection and appropriate storage	^23–25^
(2) Anti-coagulants	The effects of anti-coagulants can present themselves in a number of ways in translational research. Many cardiovascular patients are administered drugs such as heparin or warfarin, which not only affects blood clotting but can also interfere with downstream molecular applications such as cDNA synthesis Anti-coagulant agents including EDTA and citrate are also used in the preparation of plasma, serum or white blood cells	^26^ ^,^ ^27^
(3) Pre-processing interval	The time between sample extraction and processing (in the case of plasma, serum or white blood cell isolation) or storage (e.g. freezing cardiac samples)	^28^
(4) Processing method	The protocol used for extraction of plasma, serum or white blood cells isolation	
(5) Samples storage	Receptacles for liquid biopsies and solid tissue can affect preservation of RNA and DNA (e.g. cryo-tubes pre-coated with anti-coagulants such as EDTA or citrate or nuclease free tubes)	^29^ ^,^ ^30^

The post-mortem interval (PMI) is a primary confounding factor affecting quality and integrity of subsequently isolated biological material, such as DNA and RNA.

Firstly, at ambient temperatures, RNA is highly labile and RNase enzymes remain catalytically active, naturally causing a decrease in RNA integrity with increased PMI. Evidence also suggests that not all RNAs are created equal, some being more susceptible to degradation.^31^

Secondly, one could envisage stress-associated gene programmes, such as those induced by hypoxia, to be activated in the early PMI period. These two main influences can greatly affect the outcome of transcriptome analysis in CVD.

For extraction of tissues from sudden-death victims, the exact PMI is not always easy to determine and will inevitably not be consistent between samples. This is a common means by which tissues from ‘healthy/control’ individuals are collected. PMI in this case is usually determined by a pathologist based on physical observations of the corpse such as body temperature and *rigor mortis*. What’s more, heart tissue collected during procedures involved in treatment and investigation of cardiovascular disease, such as endomyocardial biopsies, valve replacements or heart transplants, is handled very differently, generally placed into cardioplegic solution followed by appropriate and rapid storage.

Significantly, an analysis of GTEx (Genotype-Tissue Expression project) tissues and expression data of different human tissues with PMIs ranging from 1 to 27 hours and good quality RNA-seq data is available. A systematic analysis showed that cardiac and skeletal muscle displayed the greatest influence of PMI on the transcriptome, with over 850 genes significantly differentially expressed depending on PMI.^23^

Comparing the transcriptome profiles between diseased and healthy heart samples, the vastly different and heterogeneous PMIs and handling protocols will influence the findings. In addition, there are no published guidelines set by standardisation bodies or networks of experts presenting consensus procedures in sample attainment, handling, or storage. A first step to improve reproducibility should be a detailed description of sample harvesting and handling in all scientific publications, information that is often missing.

The effect of the pre-processing time, as well as RNA integrity on the global depth of RNA-seq and on the outcome of gene expression analysis, was evaluated in only two studies of human peripheral blood mononuclear cells and human tissue (human placental samples).^32^^,^^33^ RNA integrity notably affected the results of gene expression analysis.

The European Commission has acknowledged a need for international unity in governance and coordination of clinical biobanks.^34^ Clinical biobanks need to adhere to minimum information requirements as set out in the Minimum Information About BIobank Data Sharing (MIABIS) tool developed by the EU BBMRI-ERIC research infrastructure.^35^

In conclusion, careful and correct collection, storage and sharing of information on pre-clinical variables as well as RNA integrity, evaluated by the gold standard RIN value, will enable consideration of these elements in translational cardiovascular research. These factors can then be incorporated as covariables in analysis platforms to identify independent gene expression changes.

## 3. Cell-type-specific vs. whole tissue transcriptomic changes

**Figure 1:**
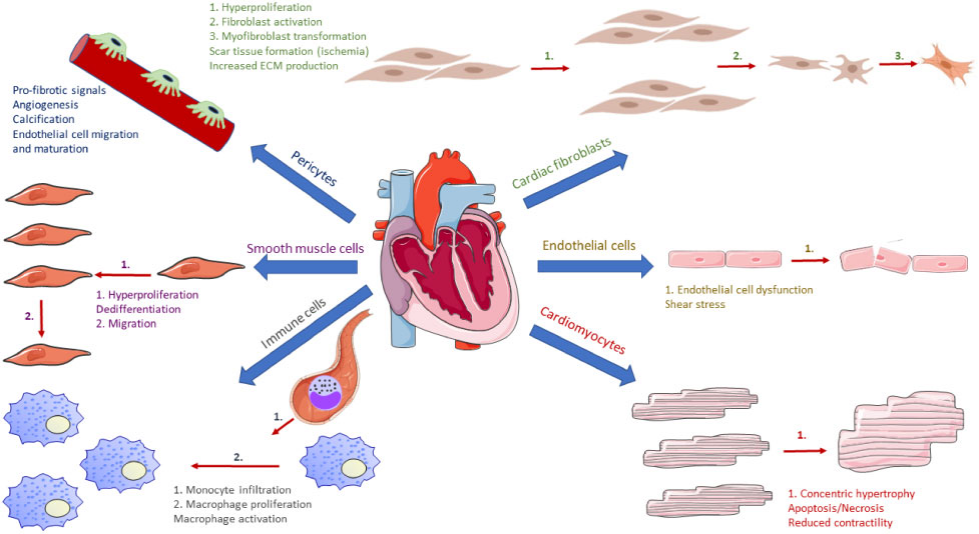
Depiction of the major cell types of the mature mammalian heart along with examples of ways cellular morphology and function can change in disease. Numbered arrows refer to the processes numbered in the text. Text and numbering is colour-coded according to cell type. From far-right clockwise: endothelial cells, cardiomyocytes, immune cells, smooth muscle cells, pericytes, and cardiac fibroblasts.

The heart is a highly heterogeneous organ, composed of at least six major cell types. The relative composition of the mammalian heart is a debated area, but it is generally accepted that CMs, cardiac fibroblasts (CFs), and endothelial cells (ECs) are the dominant cell types.^36^^,^^37^ Other minor cell types include smooth muscle cells, pericytes, and macrophages. Relative cell type composition changes through development, ageing, and disease, with proliferation of most cell types occurring during mammalian development and growth. Conversely, ageing and disease are characterized by CM hypertrophy, attrition and apoptosis, by CF hyperproliferation and transformation into myofibroblasts, and by intramyocardial infiltration of immune cells. *Figure 1* depicts the composition of the adult heart and how different cell types are typically altered in response to chronic pathological stimuli.

Alike, the vasculature is composed of smooth muscle cells, ECs, pericytes as well as a significant layer of connective tissue and elastic fibres. Remodelling of the vessel bed composition, cellular biology, morphology, and structure occurs in injury and atherosclerosis.^38^

Considering all the aforementioned factors and substantial remodelling of cardiac cellular composition in disease, transcriptomic data from whole heart tissue are not representative for a true comparison of changes within a particular cell type and make translating bulk molecular changes to biological function difficult. The resulting data sets represent only composite changes across the constituent cell types for that sample, often diluting changes that are of relevance in cellular and functional remodelling.

A number of established protocols exist for isolation of CMs, CFs, ECs and immune cells from fresh heart tissue from animals and humans. These methods are centred around enzymatic digestion, often involving specialist equipment such as Langendorff apparatus for lengthy periods of aortic and coronary perfusion. Achieving high quality, high purity and consistent cell preparations proves variable between protocols, different laboratories as well as between species, samples and experimental conditions.

One further significant caveat to isolation and separation of different cell types by enzymatic digestion is that fresh tissue is required, which needs to be maintained in cardioplegic solution since the point of extraction. Not only in some cases is access to fresh tissue impractical, such as in the case of biobanked human material, but the time of the preparations undertaken at ambient temperatures and conditions are conducive to DNA synthesis, transcription, RNA degradation as well as epigenetic modifier activity during the preparation time.^39^

Furthermore, as previously indicated, anti-coagulants such as heparin administered during extraction of the heart as well as anti-thrombotic medication in patients, have been shown to interfere with cDNA synthesis reactions used in RT-qPCR or RNA-seq library preparations.^26^

Variations on these methods for fresh CM and CF isolation have been developed with the aim of optimizing purity, cellular integrity, and minimizing the need for anti-coagulants and complex apparatus, including through direct needle perfusion of the extracted heart *ex vivo*.^40^^,^^41^

All confounding factors and circumstances considered, further approaches have been formulated to obtain cell type-specific gene expression or epigenomic information. Whilst intact living cells can only be isolated from fresh cardiac tissue, nuclei can be extracted from frozen tissue. Fluorescence immunolabelling of cell-type-specific nuclear antigens followed by flow cytometry enables collection of cell-type-specific DNA and nuclear RNA. Examples include the use of pericentriolar material-1 (PCM-1), nuclear-retained phospholamban, or troponin T for CM nuclear isolation.^42–45^ A further benefit of this approach is that all steps can be performed at ice-cold temperatures and with addition of inhibitors of RNA Pol II and epigenetic mediators, such as sodium butyrate to suppress histone deacetylase activity, thereby reducing preparation-associated transcriptome and epigenetic remodelling.

It should be noted that nuclear transcriptome data give a different readout to cellular or bulk tissue RNA analysis. Firstly, 90% of the total RNA in the cell is in the cytoplasm as mature processed RNA species. The nucleus is home to pre-processed RNA such as pre-mRNA, pri- and pre-miRNAs, and nascent lncRNA transcripts. In addition, nuclear-localized RNA species, such as lncRNAs, will be overly represented in the nucleus. Nuclear content gives a readout of more of the nascent transcription rates whereas cellular RNA is reflective of additional post-transcriptional regulation such as miRNA mechanisms and mRNA stability.^46^^,^^47^ Provided a pure nuclear population is isolated, transcripts from the 37 mitochondrial encoded genes will also be depleted. That said, direct comparison of results of global tissue or cellular vs nuclear RNA in a variety of models and species found strong correlations between the data sets from different origins.^48^

One further approach to deducing the relative contribution of cell types in big transcriptomic data from bulk tissue or a mixed cell population is through bioinformatic deconvolution using established knowledge drawn from gene expression profiles of the individual composing cell types. One such example of where this strategy is employed very effectively is with whole blood specimen, where gene expression profiles of blood cell types—predominantly white blood cells—and sub-types are well described.^49^ An example of one such pipeline that has been developed for this purpose is Decon2.^50^

Major advantages of this strategy include minimization of confounding factors involved in tissue separation and specific cell or nuclear isolation and sequencing, with the resulting data representing the relative cell population and tissue transcriptome close to that in vivo, provided appropriate extraction and storage of the biospecimen.

That said, published examples of where bioinformatical deconvolution in this manner has been successfully employed in cardiovascular tissues are lacking. One probable reason for this is that transcriptional dysregulation is commonly seen in the context of disease, with cells of the cardiovascular system ectopically expressing genes atypical of their differentiated cell state. For example, CMs have been shown to express extracellular matrix protein in pro-fibrotic conditions and as fibroblasts are activated towards a myofibroblast state, express contractile fibres.^51^^,^^52^ Leading up to the following section of this review article, with further single-cell RNA-sequencing (scRNA-seq) data being generated from cardiovascular tissues and shared through data archives, we can hope that bioinformatic deduction of the relative contribution of cell types and sub types to bulk tissue expression changes will soon evolve in the cardiovascular field.

## 4. Single-cell transcriptomic analysis

The advent of scRNA-seq technology provides an unparalleled opportunity for high-resolution profiling of the global transcriptional signature of individual cells in models of disease and human tissue. Since the seminal report of transcriptome sequencing at the single-cell level of a mouse blastomere a decade ago, there has been an early exponential rise in studies applying this technology in the cardiovascular system.^53^ ScRNA-seq permits us to catalogue the cellular composition of a tissue and to classify cell and transcriptional heterogeneity and plasticity under different conditions in an unbiased fashion. Therefore, scRNA-seq holds great promise as an accessible technology that can provide pivotal in-depth insight into the molecular regulators associated with CVD and regeneration.

Analyses of bulk tissues produce signals that are an average across several cell-types in the tissue and so are unsuited to defining the role of individual cells. However, scRNA-seq allows the characterization of gene expression changes in individual cells and therefore an opportunity to deconvolve both cellular and disease-induced heterogeneities that serve biological functions.

In the heart, several papers reporting single-cell transcriptional profiling of cardiac lineages from adult mice have emerged over the past two years. In a landmark study, single-cell transcriptomic was applied to map changing cell subpopulations in the healthy and injured mouse heart 3 days after ischaemia-reperfusion.^54^ This identified *cytoskeleton-associated protein 4* (*Ckap4*) as a specific marker of activated CFs following injury. In another study, Forte and colleagues used scRNA-seq to characterize the dynamic transcriptional changes in non-CM cells at multiple timepoints following MI, identifying the occurrence of an early myofibroblast response as a predictor of cardiac rupture.^55^ A high-resolution single-cell gene expression atlas of isolated mouse cardiac ECs defined 10 transcriptionally-distinct, heterogeneous cardiac EC states resident in the adult mouse heart at a time of peak neovasculogenesis following ischemic injury, and provided a rich resource of targets with a potential role in vascular regeneration of the injured myocardium.^56^ Sex-dependent transcriptional differences in cardiac cell subtypes were also shown, which may provide fundamental insight into the molecular cues that underpin sexually dimorphic responses to cardiac injury.^57^ In the last year, studies have been published using single nuclear and cellular sequencing from cardiac tissue describing the transcriptional landscape and heterogeneity in different chambers of the heart and between sexes in the context of heath and heart failure (HF).^58^^,^^59^ ScRNA-Seq has also revealed the extent of immune system activation in a mouse model of pressure overload-induced HF.^60^

Outside the heart in the peripheral vascular setting, a number of studies have reported scRNA-seq of cells in blood vessels, for example in healthy and atherosclerotic mouse tissue.^38^^,^^61^^,^^62^ This has characterized the transcriptional signatures of specific vascular lineages and subpopulations, including the molecular profiling of a potential endovascular progenitor cell, with a large cluster of ECs expressing clear mesenchyme-associated genes even in healthy aorta. Two independent relatively large-scale studies have used scRNA-seq to map the transcriptional landscape associated with pluripotent stem cell commitment to vascular lineages, with a focus on ECs.^63^^,^^64^ McCracken sequenced over 100,000 single cells at several time-points during the differentiation protocol to identify many previously unknown transcription factors that are activated upon commitment of pluripotent cells to the endothelial lineage.^64^ This knowledge may be key for future studies aiming to interrogate specific facets of vascular biology such as specification to distinct endothelial types, perhaps even organ-selective endothelial types.

See and colleagues applied single nuclear sequencing technology (snSeq) to identify both coding and non-coding RNA targets in CMs isolated from the left ventricles of healthy and failing hearts in mice and humans.^65^ Core markers were defined, including non-coding RNAs, expressed by healthy CM subpopulations and common to both mice and humans. This study further characterized transcriptional heterogeneity in stressed CMs and identified lncRNA candidates, including *Gas5* and *Sghrt,* as regulators of gene expression networks associated with CM proliferation.

**Figure 2:**
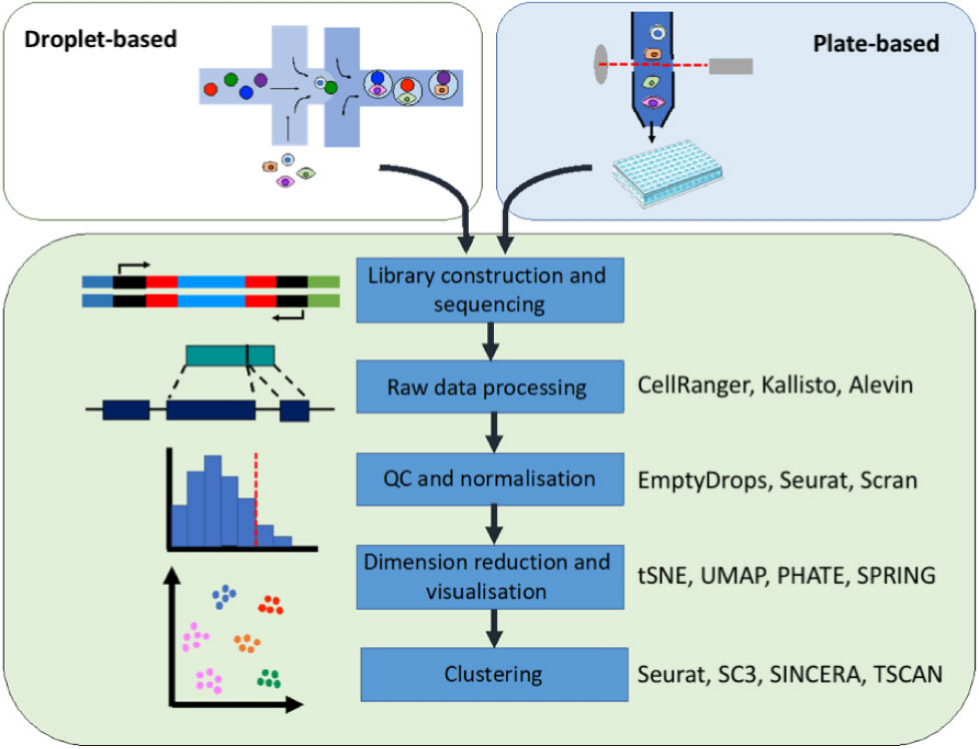
Schematic of a typical scRNA-seq workflow: single cells are separated and sequencing libraries generated using either droplet or plate-based scRNA-seq platforms. Raw sequencing data are processed and aligned to the reference genome to generate count matrices. Following quality control (QC) and normalisation, dimension reduction is performed to facilitate visualisation in low dimensional space. Subsequent clustering is performed to group cells by their similarity in transcriptional profile. Frequently used bioinformatic tools for each of these steps are indicated on the right side.

**Figure 3:**
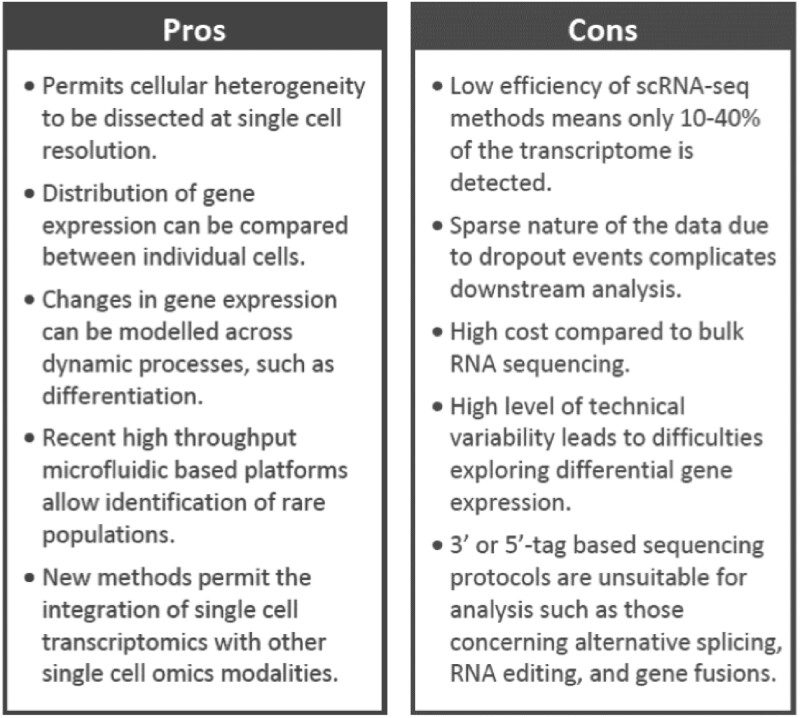
Advantages (Pros) and challenges (Cons) of single-cell sequencing technology.

The application of single-cell technology has highlighted dynamic transcriptional plasticity and heterogeneity, including classification of previously unreported or rare cell subpopulations. However, discrepancies are already apparent. For example, Farbehi *et al.* found widespread expression of *Ckap4* across virtually all cardiac stromal populations in their analyses, dissimilar to the findings of Gladka and colleagues that *Ckap4* was specific to injured and activated CFs.^54^^,^^66^ This can likely be explained by technical and methodological discrepancies such as differences in the number of cells sequenced, read depth per cell, thresholds applied to filter out background ‘noise’, and a lack of consensus over a standardized workflow and tools for analysis, as described in *Figure 2*. Great care must be taken when interpreting individual datasets following stand-alone analysis, and recent studies have emphasized the benefit of aligning multiple independent datasets using integrated bioinformatics pipelines, including from different species, as a means to identifying more robust and conserved targets. Archiving of raw sequencing datasets in open-access repositories may help to promote reproducibility and reduce potential issues caused by technical and biological discrepancies. An overview of the pros and cons of scRNA-seq technology is displayed in *Figure 3*.

Although several statistical frameworks exist for differential gene expression (DE) analysis for the bulk RNA-seq, methods for scRNA-seq are just emerging.^67^^,^^68^ The real challenge in sn/scRNA-seq DE analysis is to take into account particular characteristics of scRNA-seq which include low library sizes, high noise levels, and a large fraction of dropout events.^69^ In addition, the stochasticity of gene expression in cells results in multimodality of gene expression across different cells.^70^ To address these challenges, several single-cell-specific methods have been developed. One example is that of model-based analysis of single-cell transcriptomics (MAST), which proposes to develop a generalized linear model to take into account bimodal distribution arisen as a result of either detectable transcripts or non-detectable genes that may have arisen because of stochastic dropout events.^71^ Although MAST has been evaluated to be one of the best performing statistical frameworks for detecting DE genes for single-cell datasets, DE analysis of scRNA-seq data remains to be a challenge, as the tools developed for scRNA-seq focus to model either dropout events or multimodality but not both.^70^

The fast-paced evolution of snRNA-seq technology, and emerging applications such as snSeq of frozen tissues, will undoubtedly continue to provide critical new insights into cardiovascular homeostasis and disease processes that then inform critical proof-of-concept mechanistic studies. To complement the information obtainable by these techniques based on tissue disaggregation, another emerging application of next-generation sequencing is that of spatial transcriptomics. This method is an expansion on in situ hybridization into an unbiased high throughput method to analyse the presence and abundance of transcripts in a two- or three-dimensional context in complex tissues. One such example, Tomo-Seq (RNA Tomography for Spatially Resolved Transcriptomics), is able to obtain a genome-wide gene expression signature with high-spatial 2-D resolution in a mouse model of myocardial infarct (MI) spanning from the infarcted area to the remote area to identify new regulators of cardiac remodelling as well as in a zebrafish model of cryoinjury to identify mediators of cardiac regeneration.^72^^,^^73^ Other newly established methods include solid-phase bar-coded RNA capture and so-called 3D-Cardiomics from which a transcriptome map has been constructed from microdissections of the healthy mouse heart.^74^ One limitation to be noted is that, to eliminate genomic DNA incorporation into library construction, current methods for spatial transcriptomics rely on polyA capture and detect mature mRNA species only.

## 5. RNA splicing and circular RNAs

Most mRNAs and lncRNAs generated in mammalian cells are composed of exons separated by introns. Certain introns are spliced-out constitutively, but many splicing signals are not used by all cells nor in all circumstances, leading to alternative splicing (AS). AS allows expansion of the genome to generate a diverse array of functional RNA transcripts from a single gene. Being estimated to occur in about 90% of human genes, AS greatly increases the coding potential of the genome.^75^ Of note, AS can generate not only different isoforms of the same protein, but also functional non-coding RNAs. Another example of splicing is that of the aforementioned 3’- to 5’-back-splicing-generating circRNA species.^12^ The circRNA landscape expressed in the human and mouse heart as well as in differentiating CMs has revealed a large proportion of gene loci from stable detectable circRNAs.^76^ The most abundant of these, *circSlc8a1-1*, was found to act as a *miR-133a* competing endogenous sponge, and its inhibition in pre-clinical pressure-overloaded models attenuated the pathological cardiac hypertrophic response.^77^ Outside of the heart, circ_Lrp6, a circRNA enriched in vascular smooth muscle cells, regulates miRNA-145 function through acting as a sponge in vascular smooth muscle cells.^78^

**Figure 4:**
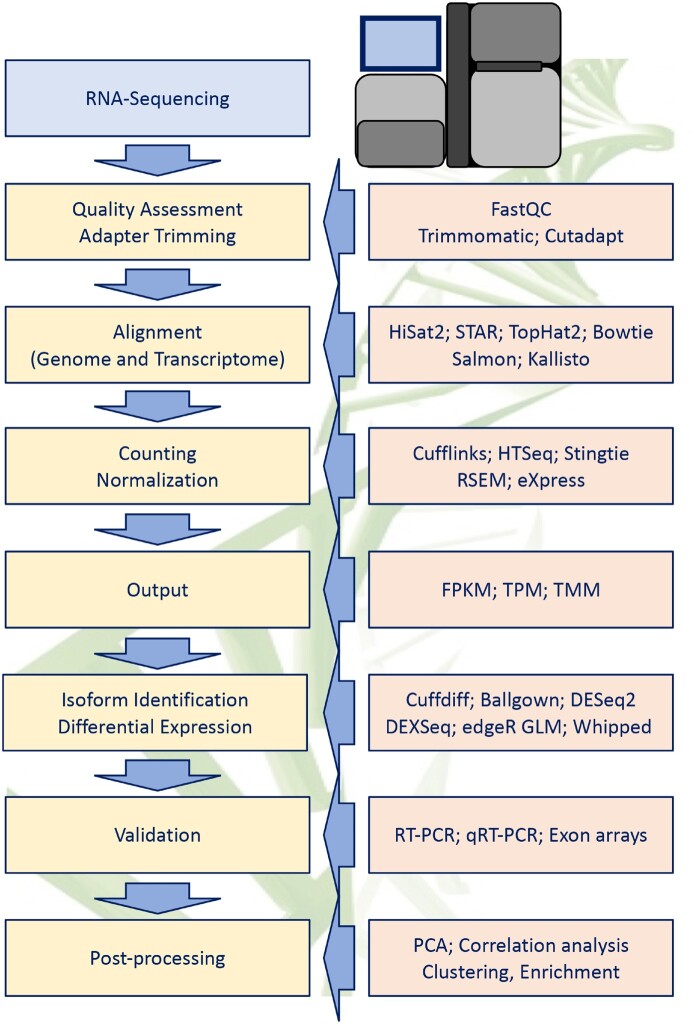
Schematic of typical alternative splicing (AS) analysis workflow using from RNA-seq data. Frequently used bioinformatic tools for each are indicated on the right side of each step.

It has been known for a long time that transcripts that are important for cardiac homeostasis and function can be aberrantly spliced in human heart disease, but the magnitude of mis-splicing events has only become evident in the past decade. Widespread use of microarrays and RNA-seq techniques has established a robust association of altered AS of pivotal cardiac genes, including many sarcomeric and ion channel genes, with cardiac hypertrophy, dilated cardiomyopathy (DCM), and HF.^79^^,^^80^ An outline for the workflow for analysing splice variants from RNA-seq data with examples of computational tools available for each step is shown in *Figure 4*.

The first generation of transcriptomic analyses used exon-microarrays to identify splicing events, and, more recently, RNA-seq has revealed the pervasiveness of AS in an unbiased manner.^79^^,^^81^ AS regulation is mediated by the interaction between *cis*-elements around the splicing site in both introns and exons, and *trans*-acting RNA-binding proteins (RBPs) that bind to specific *cis*-elements. RBPs are dynamically regulated with a variety of mechanisms and can promote or inhibit the splicing of a specific intron. A subset of RBPs regulating AS in the heart have been identified, including RBFOX-1 and -2, MBNL1 (muscleblind-like protein 1), CELF1 (CUGBP Elav-Like Family Member 1), and RBM-20 and -24 (RNA Binding Motif Protein 20 and 24).^75^ Evidence of the importance of these RBPs for heart physio-pathology derives mostly from genetic engineering of mouse models and cell culture experiments. Indeed, so far, only mutations in RBM20 have been shown to be associated with cardiomyopathy.^82^

RBM20 mutations cause an early-onset and clinically aggressive form of familial DCM, associated with aberrant inclusion of specific exons in the Titin (TTN) transcript.^83^ This, in turn, results in the expression of very large and compliant TTN isoforms affecting the physiological heart passive stiffness.^83^

TTN is a very large multi-exon gene encoding many transcript variants regulated by still poorly understood AS events. Transcriptomic analysis of cardiac-specific knockout mice indicates that TTN is also one of the direct splicing targets of murine Rbm24.^84^ It remains an open question whether human RBM24 is a cardiomyopathy-associated gene, since no mutations in the three main isoforms of RBM24 have been identified in DCM patients.^85^ Of note, specific circRNAs originating from TTN are also absent in RBM20 null mice.^86^ Another RBP, Quaking, also regulates the formation of TTN-circRNAs, that are likely mediators of Quaking cardio-protective effects.^87^

RBPs can engage in antagonistic actions, as observed for CELF1 and MBNL1. While CELF1 decreases during heart development, MBNL1 increases.^88^ Manipulation of these RBPs in the adult heart replicating their levels in the embryo results in reactivation of the embryonic splicing pattern.^88^ Indeed, re-expression of a foetal splice variant programme is a phenomenon characterizing most cardiomyopathies.^81^

In particular, in myotonic dystrophy type 1 (DM1), extensive AS changes result from the CUG expansions in the DMPK gene characterizing the disease.^89^ These triplet-repeat RNAs accumulate in the nucleus, acting as molecular sponges and altering the function of MBNL, CELF1, and other splicing factors, leading to re-expression of foetal isoforms in adult tissues, including the myocardium. Accordingly, after respiratory failure, cardiac arrhythmias are the most common cause of death in DM1 patients.^90^

Another mechanism of AS regulation is made possible by splicing occurring co-transcriptionally for most genes, allowing the interaction between the splicing and the RNA-synthesis machinery.^91^ Thus, AS can be affected by RNA transcription kinetics, as well as by chromatin modifications and structure.

Many computational methods have been developed to analyse RNA-seq datasets at exon level. All these methods rely on reads spanning each exonic junction, to estimate an abundance ratio between the two spliced isoforms (exclusion and inclusion isoforms), followed by a statistical test to assess the significance of the observed difference in splicing. Although implemented with a variety of statistical frameworks, all available methods suffer from high uncertainty for the AS events sampled with low sequencing coverage. High sequencing depth is necessary to obtain a representative picture of the gene expression pattern at isoform level. The situation may be even more critical for circRNAs: these RNA species are devoid of a 3’-end and published datasets deriving from poly-A positive RNA cannot be used for their detection.

A further, so-called third-generation sequencing technology, which may be beneficial in the identification of novel transcript isoforms, is that of long-read sequencing. Such long-read sequencing technologies have been developed by, for example, Pacific Biosciences PacBio or single-molecule, real-time (SMRT) sequencing and Oxford Nanopore Technologies (ONT) and are able to sequence full-length transcripts in excess of 10 kb in length. The advantages of long-read sequencing over classic short read include lower error rates in mapping, and reduce amplification bias, easier accurate assembly of regions of the genome comprising repetitive sequences, and *de novo* gene assembly for poorly annotated genomes and genes as well as valuation of copy number variants. This has been reviewed extensively previously.^92^ Long-read sequencing has proven an effective strategy to identify novel transcripts and super repeat regions in very large muscle structural genes such as TTN and Nebulin (NEB), the biological relevance of which in cardiac and skeletal muscle is yet to be determined.^93^

Although there has been significant progress in the characterisation of isoform-level transcriptomics and the discovery of the regulatory aspects of AS networks, demonstration of the functional consequences of the expression of different isoforms of individual genes is still scarce. Therefore, understanding the physio-pathological relevance of AS is one of the biggest challenges of CVD functional genomics.

## 6. Subcellular compartmentalisation of RNA

Unequal cellular distribution is characteristic of the majority of RNA species. Concentrations of RNAs at specific locations are highly associated with their functionality. A prime example is the transport of mRNAs from their site of production, the nucleus, to the cytoplasm, where they are actively translated.^94^ Binding to ribosomes creates a microdomain enriched in mature mRNAs. In addition, subcellular concentrations of ribosome-bound mRNAs provide a means of controlling local protein production, allowing creation of functional protein gradients. In turn, localized protein translation regulates key cellular processes such as specification and differentiation. In the heart, Mbnl regulates mRNA localisation via binding to 3’UTRs of transcripts encoding membrane proteins, consistent with the Mbnl localisation at the periphery of the cell.^88^ In CMs, YB-1, a single-strand nucleic acid-binding protein that regulates expression of proteins associated with tissue repair, accumulates within the polysomes in the sarcoplasm, close to the intercalated disk, resulting in increased smooth muscle actin deposition in cardiac sarcomeres at this location. These examples suggest translation occurs at pre-specified sites to favour targeting of proteins at relevant locations.^95^

MiRNA processing and maturation is an example of regulated changes in localisation. MiRNA genes are genomically encoded individually or as clusters, intergenically or intronically to protein-coding genes.^5^ Primary miRNA transcripts are synthesized in the nucleus, processed by Drosha, exported to the cytoplasm and cleaved by Dicer to produce mature miRNAs. Then, miRNAs are loaded in a silencing complex (RISC) together with Argonaute proteins. This allows pairing to and targeting mRNAs to silence expression. Most mature miRNAs demonstrate therefore cytoplasmic enrichment. Moreover, processing bodies (P-bodies) are cytosolic organelles, which are the primary sites of miRNAs activity. Interestingly, the RISC is required for maintaining P-body integrity.^96^ RISC is also associated with polysomes, suggesting that miRNA-mediated repression is initiated in the rough endoplasmic reticulum. Altogether, miRNAs are concentrated in the cytoplasm at specific *foci*, each representing a functional step in the silencing pathway leading to mRNA degradation. On the other hand, the miRNA-loaded RISC can shuttle back into the nucleus, where it can exert additional regulatory function on transcription and splicing in the nucleoplasm or even in the chromatin. Finally, mature miRNAs are also found in mitochondria.^97^ They originate from the cytoplasm, but can also derive from the mitochondrial genome. Their actions involve fine-tuning of various mitochondrial functions via regulating the expression of respiratory chain subunits, and thereby mitochondrial electron transport and oxidative phosphorylation.

There is a further emerging role for Argonaute proteins in the formation of RNA-induced transcriptional activation (RITA) complexes in association with small activating RNAs (saRNAs). RITAs translocate into the nucleus to enhance gene expression. Description and understanding of endogenous saRNAs in cardiovascular disease are yet to be unveiled, and there may be a potential to harness saRNA biology as a therapeutic strategy.^98^

Molecular mechanisms controlling the correct targeting of lncRNAs to their ultimate site of action are intrinsic parts of their functionality. Nuclear-enriched lncRNAs are mainly involved in transcriptional and epigenetic regulation through cis- or trans-mechanisms, acting at their site of transcription or via travelling to remote locations in the genome respectively. A large number of lncRNAs are also exported to the cytoplasm. Visualisation of nuclear lncRNAs using RNA fluorescent *in situ* hybridisation identifies lncRNAs either in discrete nuclear *foci* or more homogeneously in the nucleoplasm. *Foci* of chromatin-bound lncRNAs reflect active lncRNAs transcription, lncRNAs that remain tethered to their own locus or accumulation at target loci, for instance through formation of RNA-DNA triplexes, a mechanism of lncRNA-mediated regulation of gene expression. Moreover, lncRNAs are observed in other compartments such as nuclear speckles and paraspeckles, organelles implicated in mRNA processing. In addition, lncRNAs can also be nuclear and cytoplasmic, or purely cytoplasmic.^99^ In this vein, lncRNAs that act as decoy for miRNAs in competing endogenous network are usually found in the cytoplasm, the site of miRNA-mediated post-transcriptional regulation. This is also the case for lncRNAs directly interacting with mRNAs for controlling their stability and degradation.

Surprisingly, despite the fact that lncRNAs are not translated, many lncRNAs are found associated with ribosomes in polysome profiling experiments. As mentioned previously, this might reflect a role for lncRNAs in translation regulation or micropeptide production.^16^^,^^17^ Last but not least, lncRNAs are found in exosomes suggesting a role for lncRNAs in cell-to-cell communication.^100^

## 7. Peripheral blood

Mechanistic studies support peripheral blood RNAs as regulators of CVD. Since peripheral blood is easily accessible, it is a promising source of biomarkers for personalized healthcare in areas with room for improved diagnosis and treatment (e.g. HF, myocarditis, valvular disease progression, and cardiogenic shock). The first studies on circulating miRNAs in cardiovascular disease focused on their diagnostic potential. Plasma levels of miR-1a and miR-133a/b were among the first miRNAs identified to be upregulated in acute MI. Many subsequent studies postulated dynamic changes in peripheral blood miRNAs in different areas of CVD, systematically reviewed elsewhere.^101^ However, a lack of protocol standardisation, small sample sizes without validation cohorts, and heterogeneous patient populations have sometimes led to inconsistent results.

When considering studies with miRNAs in larger populations with independent validation, the largest body of evidence is available for miR-1, miR-133a/b, miR-208, and miR-499. These muscle- and cardiac-enriched miRNAs increased significantly in patients undergoing septal ablation for hypertrophy, suggesting a troponin-like biomarker potential.^102^^,^^103^ However, after adjusting for cardiac troponin T or clinical variables in independent studies, these miRNAs lost significant diagnostic or prognostic capabilities in patients with chest pain or acute coronary syndrome.^104^^,^^105^ Other miRNAs, for example endothelium-enriched miR-126, have consistently been shown to be related to platelet function making them suitable as candidates for the prediction of atherothrombotic events in primary or secondary prevention.^106–108^

Besides miRNAs, the biomarker potential of mRNAs, lncRNAs, and circRNAs was investigated after a few landmark reports in stable coronary artery disease (CAD), acute MI, and HF.^14^^,^^109^ Plasma levels of *LIPCAR* and whole blood levels of *QSOX1* and the circRNA *MICRA* have been identified and replicated as novel prognostic markers of subsequent left ventricular dysfunction in patients with MI, independent from classical prognostic markers including NT-proBNP.^15^^,^^110^^,^^111^

**Figure 5:**
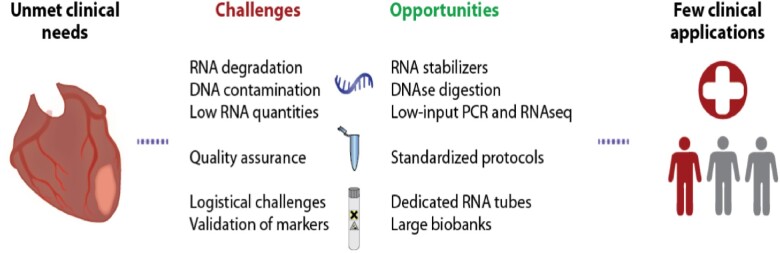
Challenges and opportunities in transcriptomic studies in peripheral blood.

Nevertheless, none of these RNAs has as yet been assessed in a large interventional clinical study to tailor therapies or improve patient outcome. Several considerations may explain the challenge to translate experimental findings from transcriptomic studies back to the bedside (*Figure 5*). These challenges include the timing of blood sampling (e.g. lifestyle-related time-of-day fluctuations as well as stage of disease onset and progression), selecting the appropriate sample type, sample handling prior to storage, RNA isolation strategy, RNA quality and correct downstream RNA expression measurement with appropriate normalisation. RNA degradation is a serious limitation of transcriptomic studies in peripheral blood samples. In a clinical setting, liquid biospecimen cannot always be extracted and stored optimally to preserve the blood transcriptome. To overcome this challenge and eliminate other pre-analytical variables, blood collection tubes containing RNA stabilizers have been developed. Even with these precautions, RNA integrity and potential genomic DNA contamination should be assessed before starting downstream applications. For cell-free RNA (e.g. in plasma or exosomes), no dedicated approach to minimize RNA degradation has been described. Heparin administration to the patient or in the sample may also result in spurious RNA expression results.^26^ Finally, the quantity of extracted RNA may also constitute a hurdle for transcriptomic analyses. The abundance of RNA transcripts can be very low in plasma, particularly lncRNAs and circRNAs, which tend to be relatively weakly expressed in blood compartments such as exosomes. However, the continuous improvement of sequencing and PCR technologies is facilitating reliable transcriptomic studies from very low amounts of starting material. The ligation-free CATS technology has been developed to prepare RNA-seq libraries from low-input RNA down to 100 picograms.^112^ Nonetheless, with these technologies, increased sensitivity might also be synonymous of increased sensitivity to detection of non-RNA species (e.g. circulating genomic DNA).^113^ A high-quality RNA is therefore necessary for transcriptomic studies from low-input RNA isolated from a small volume of plasma/serum or exosomes.

Only two RNA-based biomarkers in vitro diagnostic assays for cardiac conditions are commercially available. Corus^®^ CAD (CardioDx, Palo Alto, CA, USA) uses whole blood RNA for the diagnosis of stable coronary artery disease in non-diabetic patients, and AlloMap^®^ (CareDx, Brisbane, CA, USA) uses peripheral blood mononuclear cells for the detection of acute rejection in patients who underwent cardiac transplantation.^114^^,^^115^ Both tests have a high negative predictive value, and the latter is the only RNA test in cardiovascular disease that demonstrated effectiveness in a clinical trial.^116^

## 8. Personalized medicine/tailored approaches

As previously discussed, the revolution in deeper and faster clinical genetic and transcriptomic phenotyping is enabling us to understand disease-associated changes in each patient. This allows the opportunity for personalizing therapeutic strategies.

Transcriptome changes in the blood (cells or plasma) may help to better diagnose or determine the prognosis of our patients. Indeed, many clinical guidelines emphasize the unmet need for personalized medicine approaches. For instance, biomarker-guided cardiovascular therapy represents an interesting approach to identify the right patients, make accurate diagnostic decisions, and monitor efficacy. NcRNA signatures provide valuable molecular insight of patient phenotypes and could add to traditional markers and established clinical variables.

Short circulating miRNAs show promise for making predictions of cardiovascular patients; plasma miRNA-1254 levels predicted changes in left ventricular (LV) volumes and LV ejection fraction at 6 months after ST elevation myocardial infarction (STEMI). The value of miR-1254 to inform tailored treatment selection and monitor ongoing efficacy deserves further investigation.^117^ LncRNA biomarkers are also being investigated as novel predictive tools to monitor therapeutic effectiveness and to stratify patients. The lncRNA SENCR blood levels before the start of pioglitazone therapy allowed distinction of responders from non-responders and were found to be a better predictor of the response to therapy than other traditional clinical variables.^118^ Thus, assessment of lncRNA levels could constitute a major breakthrough in the development of personalized medicine to guide medical therapy, with important implications in terms of adverse effects and treatment costs. Another example demonstrating the potential of circulating miRNAs to predict the response to LVAD has been previously evaluated in patients with severe advanced HF. Morley-Smith *et al.* classified the clinical response to LVAD therapy based on NT-proBNP concentration at 3 months.^104^ Selected patients with a NT-proBNP above the 50th percentile were identified as ‘poor responders’ whereas patients below the 50th percentile as ’good responders’. By screening >1000 miRNAs, miR-1202 was found to be a useful biomarker to predict the response to LVAD therapy. Baseline circulating miR-1202 levels stratified the cohort into poor and good responders with greater sensitivity and specificity than baseline NT-proBNP. Thus, miR-1202 emerges as an interesting biomarker to select LVAD therapy or alternative interventions, such as urgent cardiac transplantation.^119^

**Figure 6:**
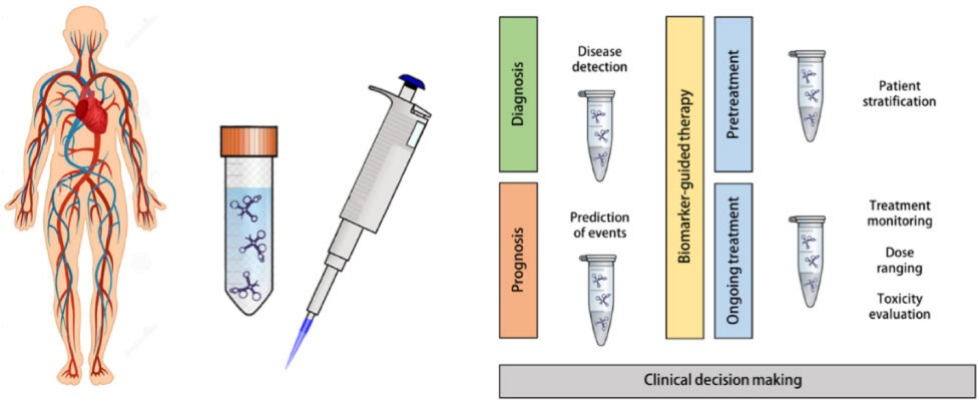
Potential clinical application of circulating non-coding RNAs in personalized cardiovascular medicine.

These examples illustrate that personalized medicine approaches show promise in becoming a reality in the near future. The rise of new technologies such as global and single-cell sequencing, the use of artificial intelligence, and machine-learning concepts will lead to tremendous new discoveries and clinically useful applications and finally also to better and safer therapies (*Figure 6*).

## 9. Brief conclusion

Recent breakthroughs in deep-sequencing technology have enabled us to delineate transcriptomes, genomes, and epigenomes from individual cells and cell types in complex heterogeneous organs and tissues of the CV system and unveil novel types, low or rarely expressed ncRNA transcripts.

With the rapid speed and reduced costs of DNA and RNA-sequencing, we are now on the brink of being in a position to fully characterize each individual patient at the molecular level. To fully realize personalized medicine, we need to bridge interdisciplinary gaps to address the challenges posed by a deeper insight into the transcriptome regulation in the cardiovascular system, and to create unity and standardisation of methodologies: from sample preparation to bioinformatics analysis to interventional studies, for ultimately translating the findings into high-impact outcomes for public health.

## Notes

This article is dedicated to the memory of Dr Paola Fuschi of the Molecular Cardiology Laboratory, IRCCS Policlinico San Donato Milanese, Italy. Dr Fuschi’s contribution to this manuscript, the scientific field, and to the lives of colleagues, friends, and family is remembered.
